# Aqueous Extract of *Siraitia grosvenorii* Alleviates MAFLD by Modulating Metabolism and Maintaining Gut Homeostasis in High-Fat Diet Fed Mice

**DOI:** 10.3390/foods15071241

**Published:** 2026-04-05

**Authors:** Hong Li, Zhongzhen Zhao, Yiming Ding, Weixian Shao, Yu Zhou, Junxiu Li, Zailin Liang, Bin Peng, Fusheng Mo, Jiao Zheng, Shengli Wei, Yuan Zhang

**Affiliations:** 1 School of Chinese Materia Medica, Beijing University of Chinese Medicine, Beijing 100029, China; 2 Agricultural and Rural Bureau of Yongfu County, Guilin 541000, China

**Keywords:** *Siraitia grosvenorii*, aqueous extract, MAFLD, lipid metabolism, oxidative stress, gut microbiota

## Abstract

Metabolic dysfunction-associated fatty liver disease (MAFLD) is the most prevalent chronic liver disease worldwide with complex pathogenesis and no approved specific therapy. *Siraitia grosvenorii* is a widely used medicinal and edible herb, yet its efficacy and underlying mechanisms against MAFLD remain poorly defined. This study explored the protective effects and potential mechanisms of aqueous extract of *Siraitia grosvenorii* (AESG) on MAFLD. Based on ultra-high-performance liquid chromatography-linear trap quadrupole orbitrap mass spectrometry (UHPLC-LTQ-Orbitrap-MS) analysis, 38 components in AESG were tentatively assigned, with tetracyclic triterpene saponins being the most abundant. In high-fat diet (HFD)-induced MAFLD mice, AESG significantly attenuated body weight gain, reduced plasma total cholesterol (T-CHO) and low-density lipoprotein cholesterol (LDL-C) levels, and dramatically decreased hepatic triglyceride (TG) accumulation from 0.0141 mmol/g in the model group to 0.0063 mmol/g in the low-dose AESG group, corresponding to a reduction of 55.00%. AESG also alleviated plasma alanine aminotransferase (ALT) and aspartate aminotransferase (AST) activities, and improved hepatocyte steatosis. Furthermore, AESG restored HFD-induced gut dysbiosis by enriching beneficial bacteria including *Akkermansia* and suppressing harmful bacteria such as *Ruminococcus*. In free fatty acids (FFA) stimulated HepG2 cells, AESG suppressed de novo lipogenesis via downregulating *Fatty Acid Synthase* (*FASN*), *Acetyl-CoA Carboxylase* (*ACC*) and *Sterol Regulatory Element-Binding Protein 1c* (*SREBP1c*), and enhanced antioxidant capacity via activating the *Nuclear Factor Erythroid 2-Related Factor 2* (*Nrf2*)/*Heme Oxygenase 1* (*HO-1*)/*Sirtuin 1* (*SIRT1*) pathway, thereby attenuating lipid accumulation and oxidative stress. In conclusion, AESG ameliorates MAFLD by inhibiting lipogenesis, improving oxidative stress, and regulating gut microbiota. These findings support *Siraitia grosvenorii* as a promising natural dietary intervention for MAFLD prevention and adjuvant therapy.

## 1. Introduction

Metabolic dysfunction-associated fatty liver disease (MAFLD) is a new term proposed in recent years [1,2]. It is used to more accurately describe liver diseases closely associated with metabolic dysfunction. Its diagnostic criteria include histopathological, imaging, or biomarker evidence of hepatic steatosis, accompanied by at least one of obesity, type 2 diabetes, or metabolic disorders [3]. The harm of MAFLD is not merely simple lipid accumulation in the liver, but a dynamic disease process that can progress from simple hepatic steatosis to hepatitis, liver fibrosis, liver cirrhosis, and even liver cancer [4]. Meanwhile, MAFLD is also a clinical disease involving multiple systems throughout the body, and its disease spectrum includes hyperlipidemia, obesity, type 2 diabetes, cardiovascular diseases, cognitive impairment, chronic kidney disease, and non-liver malignant tumors, among other extrahepatic manifestations [5,6,7,8,9]. MAFLD is a common chronic liver disease worldwide, with an estimated 25% of the adult population suffering from MAFLD globally [10], posing significant health risks and economic burdens to the global healthcare system. Currently, the treatment of MAFLD mainly focuses on early lifestyle intervention, including weight loss and physical exercise, and there are no approved and effective pharmacological treatments widely used [11,12]. Therefore, the current clinical prevention and control of MAFLD are centered on comprehensive intervention, which primarily includes maintaining a healthy weight through reasonable dietary control and regular physical activity, avoiding high-sugar, high-fat, and high-calorie diets, actively improving metabolic syndrome-related risk factors such as insulin resistance, hypertension, and dyslipidemia, while reducing alcohol intake and avoiding the misuse of hepatotoxic drugs, so as to delay disease progression and reduce the risk of cirrhosis and cardiovascular complications. However, long-term adherence to lifestyle interventions is extremely challenging for most patients, and the effects are limited. Therefore, MAFLD has become a major public health issue that urgently needs to be addressed.

Against this backdrop, natural products with a long history of edible and medicinal use have demonstrated great potential. *Siraitia grosvenorii* (Luo Han Guo, LHG), a traditional resource for both medicinal and edible purposes in China, is mainly produced in the Guangxi Zhuang Autonomous Region and is a genuine medicinal material there. It is cultivated in the typical red soil of this region, with a soil pH of 5.5–6.5 and abundant organic matter content; *Siraitia grosvenorii* prefers a humid environment but is intolerant to waterlogging, and its vegetative growth period is from April to September. In folk practice, it is often soaked or decocted with water as a solvent to make a substitute for tea. Traditionally, it has been used for preventing and treating conditions such as lung heat-induced dry cough, sore throat, and hoarseness. Modern pharmacological studies have shown that LHG also exhibits a variety of bioactivities, including regulating lipid metabolism [13], anti-obesity effects [14], improving insulin resistance [15], anti-inflammatory properties [16], and antioxidant activity [17]. Notably, these are exactly the pathological features of MAFLD [18,19,20]. Compared with synthetic drugs, the aqueous extract of *Siraitia grosvenorii* (AESG) is a complex system with multiple components, which may regulate MAFLD through multiple pathways, thereby achieving a comprehensive therapeutic effect. In addition, no reports on the toxicity of LHG have been found [21,22,23], and its safety in long-term use provides strong support for its clinical translation.

Although existing studies have suggested that LHG and its active components exert positive effects in metabolism-related diseases, there remains a significant gap in the current literature: most studies focus on the isolation and purification of a single saponin, while neglecting the holistic effect of the aqueous extract—a traditional form of medication. To further systematically evaluate the efficacy of LHG in improving MAFLD, this study used an aqueous extract—which is most consistent with traditional usage—as the research subject. A mouse model of MAFLD with hepatic lipid accumulation was established via high-fat diet (HFD) induction, and different doses of the AESG were administered to the mice by gavage. Subsequently, indicators such as body weight, blood lipids, hepatic lipids, and liver function were measured in mice from different treatment groups. Combined with observations of histopathological sections and microbiome analysis of cecal contents, this study systematically evaluated the effect of AESG intervention on HFD-induced MAFLD in mice. To further clarify the potential molecular mechanism of AESG in improving MAFLD and verify its therapeutic effect in vitro, a cellular model of MAFLD was established by free fatty acid (FFA) induction. The levels of triglyceride (TG), superoxide dismutase (SOD), malondialdehyde (MDA), and glutathione (GSH) in cells were detected to evaluate the regulatory effects of AESG on lipid accumulation and oxidative stress in MAFLD cells. Additionally, the expression levels of genes related to lipid synthesis and oxidative stress were detected by real-time quantitative polymerase chain reaction (RT-qPCR) to explore the underlying molecular pathways involved in the therapeutic effect of AESG on MAFLD.

## 2. Materials and Methods

### 2.1. Materials and Reagents

The LHG samples used in this study were sourced from Yongfu County, Guilin City, Guangxi Zhuang Autonomous Region. They were identified by Professor Zhang Yuan as the dried fruits of *Siraitia grosvenorii* (Swingle) C. Jeffrey ex A. M. Lu et Z. Y. Zhang, a plant belonging to the Cucurbitaceae family. The voucher specimen number of the LHG sample is LHG 1#, and it is deposited in the Sample Room A301, School of Chinese Materia Medica, Beijing University of Chinese Medicine.

The high-fat diet for mice (catalog number: D12109C) was purchased from Beijing Keao Xieli Feed Co., Ltd. (Beijing, China); heparin sodium, Fatty acid-free bovine serum albumin (BSA, purity 98%, Cat. No. JS244050), sterile ultrapure water, Trizol total RNA extraction reagent were purchased from Shanghai Yuanye Biotechnology Co., Ltd. (Shanghai, China); assay kits for total cholesterol (T-CHO), triglyceride (TG), low-density lipoprotein cholesterol (LDL-C), high-density lipoprotein cholesterol (HDL-C), alanine aminotransferase (ALT), aspartate aminotransferase (AST), superoxide dismutase (SOD), malondialdehyde (MDA), and reduced glutathione (GSH) were all purchased from Nanjing Jiancheng Bioengineering Institute (Nanjing, China); sodium pentobarbital was obtained from Sigma-Aldrich Corporation (St Louis, MO, USA); Oil Red O (ORO) staining solution, hematoxylin staining solution, All-in-One First-Strand Synthesis Master Mix, and Universal 2 × Realab Green PCR Fast Mixture were purchased from Beijing Lanbolide Trading Co., Ltd. (Beijing, China); 0.9% sodium chloride injection was purchased from Shijiazhuang Siyao Co., Ltd. (Shijiazhuang, China); 4% universal paraformaldehyde tissue fixative was purchased from Lanjieke Technology Co., Ltd. (Beijing, China); Sodium oleate (purity 98.0%, Cat. No. C15166694) and sodium palmitate (purity 99%, Cat. No. C16656214) were purchased from Shanghai Macklin Biochemical Technology Co., Ltd. (Shanghai, China); Cell culture-grade phosphate-buffered solution (PBS, pH 7.4) was purchased from Corning Inc. (NY, USA); DMEM high-glucose medium, Penicillin-streptomycin double antibody reagent were purchased from Gibco (MA, USA); Fetal Bovine Serum (FBS) was purchased from VivaCell, Israel; 0.25% EDTA trypsin digestion solution, 0.4% trypan blue staining solution, and cell culture-grade dimethyl sulfoxide (DMSO) were purchased from Beijing Solarbio Science & Technology Co., Ltd. (Beijing, China); Rapid cell cryopreservation solution (Cat. No. BY002P) was purchased from Shanghai Yamei Biomedical Technology Co., Ltd. (Shanghai, China); CCK-8 assay kit and BCA protein concentration determination kit were purchased from Beijing Bairui Ji Biotechnology Co., Ltd. (Beijing, China).

### 2.2. Preparation of AESG

An appropriate amount of dried LHG was weighed, crushed, and mixed with 10-fold volume of ultrapure water at a weight (g): volume (mL) ratio of 1:10. After soaking for 1 h, the mixture was decocted for 1 h and filtered through double-layer gauze. The filter residue was added with 8-fold volume of water, decocted again for 1 h, and filtered through gauze. The two filtrates were combined, and then concentrated to a concentration of 1 g/mL (equivalent to 1 g of raw medicinal material per 1 mL of concentrated solution) using a rotary evaporator at 60 °C. The concentrated solution was transferred to a −80 °C environment for overnight freezing, and then lyophilized with a laboratory-scale vacuum freeze dryer under the following conditions: vacuum level < 10 Pa, condenser temperature −55 °C, and drying time 48 h, to obtain AESG lyophilized powder. The yield was approximately 43.5%, and the powder was stored in a desiccator for subsequent experiments.

### 2.3. Qualitative Analysis of AESG

Chromatographic Conditions: Ultra-high-performance liquid chromatography-Linear trap quadrupole orbitrap mass spectrometry (UHPLC-LTQ-Orbitrap-MS) technology was used to identify and analyze the chemical components of AESG. For the separation of samples, an Acquity UPLC HSS T3 chromatographic column (1.8 μm, 2.1 mm × 100 mm) was used, with the column oven temperature set at 30 °C and the detection wavelengths set at 203 nm and 345 nm. The mobile phase consisted of 0.1% formic acid aqueous solution (Phase A) and acetonitrile (Phase B), the injection volume was 4 μL, and the gradient elution conditions are shown in Table S1.

Mass Spectrometric Conditions: The mass spectrometry system was equipped with an electrospray ionization source operating in both positive and negative ion modes. The spray voltage was set at 3.0 kV, the ion source temperature was 400 °C, the capillary voltage was 35 V, and the tube lens voltage was 110 V. High-purity nitrogen (purity > 99.99%) was used as the sheath gas and auxiliary gas, with pressures of 40 kPa and 10 kPa, respectively. Data acquisition was performed using Fourier transform high-resolution full MS scan combined with a data-dependent acquisition (DDA) MS/MS scan. A full MS scan was first carried out over the mass range of m/z 120–1800. The top 10 most intense ions were automatically selected for MS/MS fragmentation at a collision energy of −40 eV. Dynamic exclusion was enabled with an exclusion duration of 5.0 s and a mass width of ±10 ppm to improve the MS/MS coverage of low-abundance components.

Data Analysis: Relevant chemical constituent information of *Siraitia grosvenorii* was collected by searching related databases, including Web of Science, Google Scholar, PubMed, China National Knowledge Infrastructure (CNKI), and Traditional Chinese Medicine Systems Pharmacology Database and Analysis Platform (TCMSP). A self-built database of chemical constituents of *Siraitia grosvenorii* was established, which contains information such as compound names, chemical formulas, and CAS numbers. The raw mass spectrometry data were imported into Compound Discoverer (CD) 3.1 software, and standardized processing of the raw data was performed, including retention time correction, background subtraction, peak identification, and peak extraction. Molecular feature extraction was conducted on the preprocessed peaks, and the corresponding potential molecular formulas were predicted based on the accurate mass, elemental composition, and other information of the mass spectrometry data. Combined search of multiple databases was used for preliminary identification of compound structures, including Thermo mzVault local database, Thermo mzCloud online database, and the self-built database of chemical constituents of *Siraitia grosvenorii*. Compounds with an mzVault best match score of more than 80 were selected as positive candidate compounds. Further supplementary searches were performed through quasi-molecular ion peaks and MS^2^ comparison, and combined with databases such as SciFinder and ChemSpider to improve the accuracy of compound structures.

### 2.4. Animal Experiments

Forty-eight 6-week-old specific pathogen free (SPF)-grade male C57BL/6J mice, weighing 18–22 g, were purchased from Si Pei Fu (Beijing) Biotechnology Co., Ltd. (Laboratory Animal Production License No.: SCXK (Jing) 2019-0010). The mice were housed in the SPF-grade barrier environment of the Animal Experiment Center at the Liangxiang Campus of Beijing University of Chinese Medicine (BUCM), with a temperature of 20–24 °C, relative humidity of 45–55%, and a 12 h light/12 h dark cycle. Regular ventilation was provided, and the mice had free access to food and water. All experimental animal feeding and operational procedures complied with the relevant regulations of the Laboratory Animal Welfare and Ethics Committee of BUCM and were approved by the committee (Ethics Approval No.: BUCM-2023081002-3101).

To account for potential animal death or modeling failure during the experiment, the initial number of animals was appropriately increased in the model and drug intervention groups. Therefore, slight differences in the final number of animals per group were observed.

**Figure 1:**
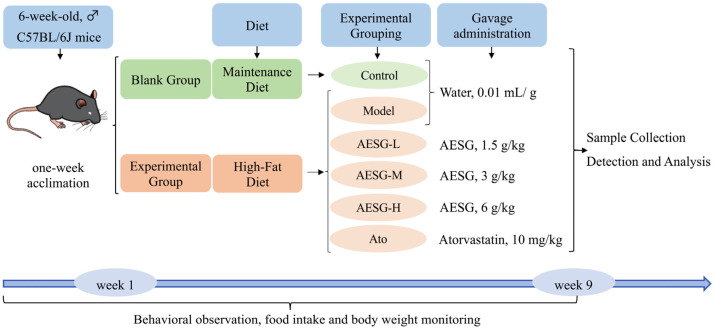
Animal experimental design.

Forty-eight SPF-grade male C57BL/6J mice were randomly divided into 6 groups (*n* ≥ 6/group): Control (normal diet + water, *n* = 6); Model (HFD + water, *n* = 10); AESG-L (HFD + 1.5 g/kg AESG, *n* = 7); AESG-M (HFD + 3 g/kg AESG, *n* = 7); AESG-H (HFD + 6 g/kg AESG, *n* = 8); and Atorvastatin (Ato) (HFD + 10 mg/kg Ato, *n* = 7). The dosage selection of AESG was based on the following: According to the Pharmacopoeia of the People’s Republic of China (2020 Edition), the recommended clinical daily dosage of *Siraitia grosvenorii* is 9–15 g. In this study, the clinical human dose was converted to the equivalent mouse dose based on the body surface area method, and three dosage groups (low, medium, and high) were established to ensure that the selected doses are both pharmacodynamically meaningful and valuable for clinical translation. All doses of AESG were calculated based on the weight of the raw medicinal material. The positive drug Ato was selected with reference to the study by Zahra Eslami et al. [24], as it can regulate fatty liver-related indicators and fasting blood glucose. After 1 week of adaptive feeding, mice in the Control group continued to receive a normal diet, while the other 5 groups were fed with HFD. The HFD was provided to mice in small, frequent portions, and fresh feed was replaced daily to prevent rancidity and deterioration. All groups of mice were gavaged at a volume of 0.01 mL/g body weight: mice in the Control and Model groups were treated with purified water, and those in the other groups were treated with the corresponding drugs. Samples were collected and processed after 8 consecutive weeks of administration (Figure 1).

### 2.5. Sample Collection and Biochemical Analysis of Experimental Animals

After the start of the experiment, the food intake of mice was recorded daily. The general conditions of mice, including whisker condition, hair gloss, urination, defecation, mental state, and activity level, were observed daily. Body weight was measured and recorded weekly.

After 8 weeks of behavioral observation and drug administration, mice in each group were fasted for 12 h with free access to water. At the end of fasting, the body weight of each mouse was measured and recorded. Blood glucose was detected using the tail tip clipping method: the tip (1–2 mm) of the mouse’s tail was cut off, the first drop of blood was discarded, and a blood glucose test strip was used to absorb the second drop for measuring fasting blood glucose levels. Mice were anesthetized by intraperitoneal injection of 1% sodium pentobarbital at a dose of 50 mg/kg. Whole blood was collected via the eyeball enucleation method and transferred into heparin anticoagulant tubes containing a small amount of liquid. The collected whole blood was thoroughly mixed with the anticoagulant, then centrifuged at 3000 rpm for 15 min at 4 °C to obtain plasma samples, which were stored at −80 °C for later use. After blood collection, mice were euthanized by cervical dislocation. The liver, spleen, kidney, epididymal white adipose tissue (WAT), and scapular brown adipose tissue (BAT) were dissected and harvested. These tissues were rinsed with pre-cooled sterile saline to remove blood contaminants, blotted dry with filter paper, and their wet weights were measured for calculating organ indices. Liver lobules and WAT were fixed in 4% paraformaldehyde solution for Oil Red O (ORO) or Hematoxylin-Eosin (HE) staining and subsequent histopathological analysis. The remaining tissues were quickly snap-frozen in liquid nitrogen and then transferred to a −80 °C refrigerator for future experiments. The levels of plasma T-CHO, TG, LDL-C, HDL-C, ALT and AST, as well as hepatic T-CHO, TG, GSH, SOD, and MDA, were detected strictly in accordance with the instructions of the commercial assay kits.

For the oral fat tolerance test (OFTT), after 7 weeks of AESG intervention, mice were fasted for more than 6 h, and blood samples were collected from the orbital sinus at 0 h. Subsequently, mice were given corn oil by gavage. Blood samples were collected from the orbital sinus at 2 h and 5 h after corn oil administration, respectively, and plasma triglyceride levels were measured.

### 2.6. Gut Microbiota Analysis

After 8 weeks of AESG intervention, blood samples were collected, and mice were euthanized by cervical dislocation. The cecal contents of each mouse were then harvested. The whole intestine was excised under aseptic conditions using a sterile scalpel. After rinsing the outer surface of the intestine with sterile water, the target cecal segment was isolated. Fresh cecal contents were collected using a sterile scalpel and forceps, placed into sterile centrifuge tubes, immediately snap-frozen in liquid nitrogen, and then stored at −80 °C for subsequent analysis. Detailed procedures for sample DNA extraction, PCR amplification, sequencing library construction, high-throughput sequencing data analysis, and statistical analysis are provided in the Supplementary Materials.

### 2.7. HepG2 Cell Experiments

The HepG2 human hepatocellular carcinoma cell line was purchased from Shanghai Fuheng Biotechnology Co., Ltd. Detailed information on the preparation of solutions related to cell experiments and the specific conditions and operating procedures of cell culture is provided in the Supplementary Materials. In this experiment, a combined induction with 400 µM sodium oleate and 200 µM sodium palmitate (with a total final concentration of free fatty acids (FFA) of 600 µM) was used to successfully establish an in vitro cell model of MAFLD. The experimental cells were divided into 6 groups with three replicates per group, and the final concentrations of each culture system were set as follows: Control group: DMEM medium only; FFA group: DMEM medium + 600 µM FFA; AESG-L group: DMEM medium + 600 µM FFA + 125 µg/mL AESG; AESG-M group: DMEM medium + 600 µM FFA + 250 µg/mL AESG; AESG-H group: DMEM medium + 600 µM FFA + 500 µg/mL AESG; positive control group (Ff group): DMEM medium + 600 µM FFA + 20 µmol/L fenofibrate (Ff). The working concentration of AESG (calculated by crude drug amount) was determined by the Cell Counting Kit-8 (CCK-8) assay.

Cells in the logarithmic growth phase of each group were collected, and the levels of TG, SOD, MDA, and GSH in each group of cells were measured strictly in accordance with the instructions of the corresponding kits, so as to evaluate the intervention effect of AESG on the in vitro MAFLD cell model.

### 2.8. Determination of Alterations in Gene Expression Levels via RT-qPCR

Real-time quantitative PCR (RT-qPCR) was performed on an Applied Biosystems™ QuantStudio™ 5 Real-Time Quantitative PCR Instrument (Thermo Fisher Scientific, Waltham, MA, USA) to evaluate the effects of AESG on the expression of genes related to lipid synthesis and antioxidant response. The specific operations are as follows: Total RNA was extracted from cells according to the instructions of the Trizol total RNA extraction reagent; mRNA was reverse-transcribed into cDNA in accordance with the operating instructions of the All-in-One First-Strand Synthesis Master Mix kit, and the cDNA synthesis system is shown in Tables S2 and S3; RT-qPCR reaction was carried out by SYBR Green method, the sequences of human-specific primers are shown in Table S4 (the primers were designed and synthesized by Beijing Dingguo Changsheng Biotechnology Co., Ltd., Beijing, China), and the RT-qPCR reaction system and thermal cycling protocol are shown in Tables S5 and S6. The detailed operating steps are provided in the Supplementary Materials. Based on the obtained Ct values, the 2^−ΔΔCt^ values were calculated, and normalization was performed using the internal reference gene *GAPDH* as the benchmark to finally obtain the relative expression levels of each target gene.

### 2.9. Data Analysis

Statistical analysis of the experimental data was performed using IBM SPSS Statistics 27, and data visualization was conducted with GraphPad Prism 10. Results are expressed as mean ± standard deviation. The Shapiro–Wilk test was used to verify the normality of data distribution. For data that satisfied both normality and homogeneity of variances, one-way analysis of variance (ANOVA) followed by the Tukey post hoc test was used to compare differences among multiple groups. For data that failed normality or homogeneity of variances, the nonparametric Kruskal–Wallis H test with the Dunn post hoc test was performed. A value *p* < 0.05 was considered statistically significant.

## 3. Results

### 3.1. Active Ingredients in AESG

**Figure 2:**
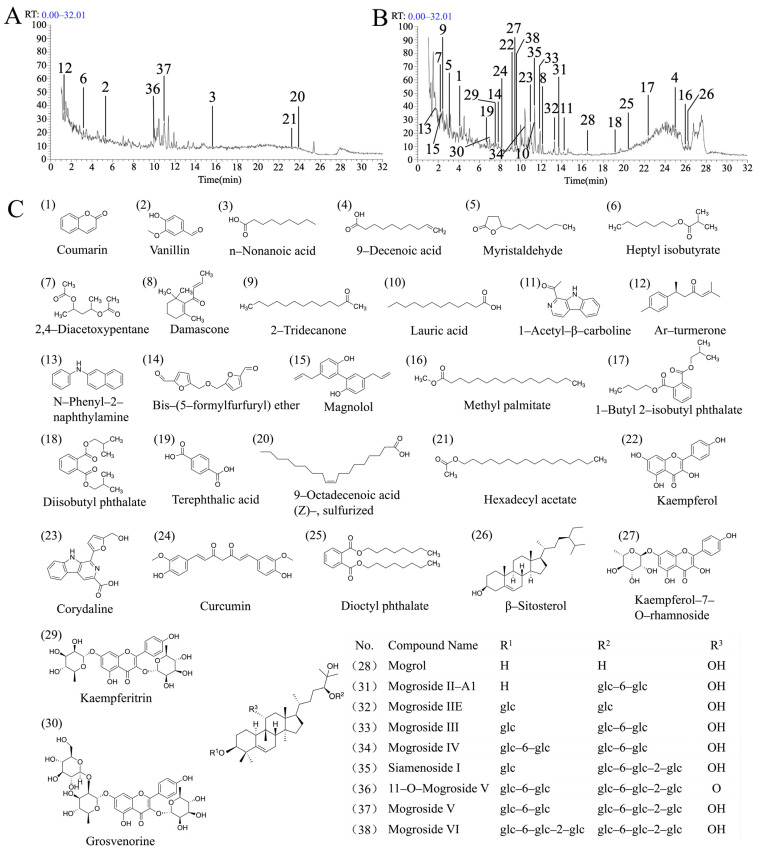
Qualitative analysis of components in AESG by UPLC-MS. (**A**) Total ion current chromatogram of AESG in negative ion mode. (**B**) Total ion current chromatogram of AESG in positive ion mode. (**C**) Structural formula of the main components in AESG. The numbers in (**A**,**B**), and the numbers in brackets in (**C**), represent the serial numbers of the corresponding compounds.

UHPLC-LTQ-Orbitrap-MS was employed to scan the AESG in both positive and negative ion modes, and the total ion chromatograms (TICs) were obtained (Figure 2A,B). Systematic qualitative analysis was performed using Compound Discoverer (CD) 3.1 software, combined with database searching, MS/MS spectrum comparison and other methods. A total of 38 chemical constituents were tentatively identified from the AESG, and the detailed information of these compounds is shown in Table S7. The chemical constituents in the AESG are abundant and diverse, mainly including phenylpropanoids, fatty acids, aromatic aldehydes, lactones, monoterpenoids, sesquiterpenoids, alkaloids, flavonoids, and tetracyclic triterpenoid saponins, etc. Among them, tetracyclic triterpenoid saponins account for a relatively high proportion and are the main category of chemical constituents in the AESG, which is consistent with the results of previously reported studies. The main structural types of chemical components in the AESG are illustrated in Figure 2C. From the compound structures, it can be observed that the aglycones of all tentatively identified tetracyclic triterpenoid saponins in AESG are mogrol.

### 3.2. Effect of AESG Consumption on Body Weight, Blood Glucose and Organs Index

**Figure 3:**
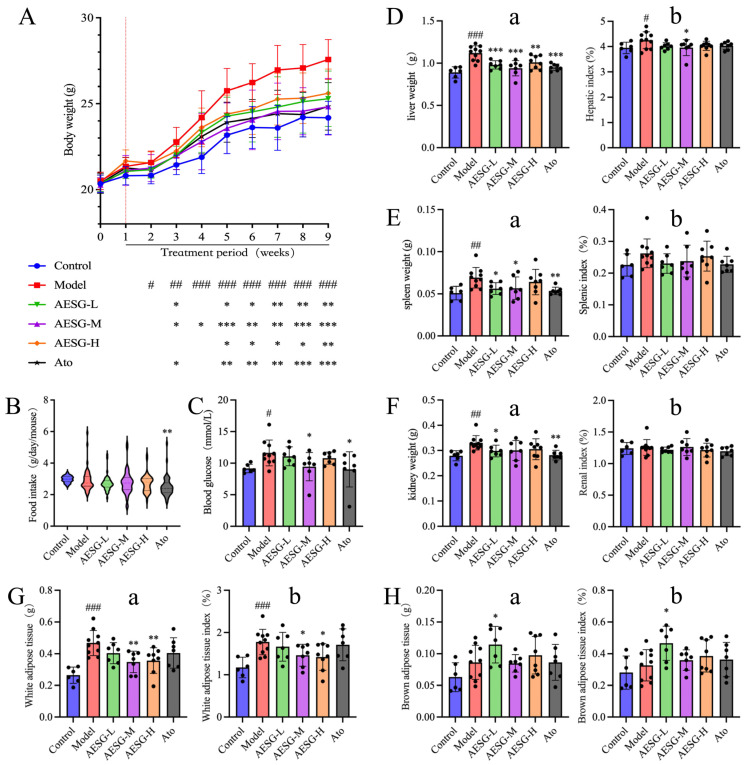
Effects of AESG on HFD mice. (**A**) Body weight of mice at different time points. Before the red vertical line: adaptive feeding; after: experimental period. (**B**) Food intake of mice. (**C**) Fasting blood glucose of mice. (**D**) Liver weight (**a**) and liver index (**b**) of mice. (**E**) Spleen weight (**a**) and spleen index (**b**) of mice. (**F**) Kidney weight (**a**) and kidney index (**b**) of mice. (**G**) WAT weight (**a**) and WAT index (**b**) of mice. (**H**) BAT weight (**a**) and BAT index (**b**) of mice. Black dots in (**C**–**H**): individual data points in the column scatter plot. Compared with the Control group, # *p* < 0.05, ## *p* < 0.01, ### *p* < 0.001; Compared with the Model group, * *p* < 0.05, ** *p* < 0.01, *** *p* < 0.001.

Before the adaptive feeding of mice, there were no significant differences in body weight among the mice in each group. During the adaptive feeding period, all mice were fed with a normal diet for the first 3 days to acclimatize to the new environment. In the subsequent 4 days, the mice in the experimental groups were given an increasingly higher proportion of HFD to gradually adapt to it, until the experimental groups were switched to a full HFD feeding regimen in the first week. As can be seen from the body weight gain trend from Week 0 to Week 1, the mice were highly sensitive to the HFD, and their body weight increased significantly after the initial intake of the HFD. Starting from Week 1, gavage administration was performed once daily while continuing the HFD-induced modeling. The body weight of mice in all groups decreased slightly due to the stress response to gavage. After overcoming the adaptation period of gavage, the body weight of mice in each group showed a gradual increasing trend. From Week 2 to Week 5, the mice exhibited a substantial increase in body weight, and the weight gain rate became relatively stable after Week 5 (Figure 3A).

There was no significant difference in food intake between the Model group and the Control group. However, with the extension of HFD feeding weeks, the body weight of mice in the Model group was extremely significantly higher than that in the Control group (Figure 3A,B). This indicates that the HFD, which is high in caloric content, led to a significant increase in the body weight of mice in the Model group. Compared with the Model group, the food intake of mice in the Ato-administered group was significantly lower. This may be attributed to reduced appetite caused by long-term administration of Ato. The decreased food intake, combined with the inherent pharmacological effects of Ato, resulted in a significant reduction in body weight in this group compared with the Model group. There were no significant changes in food intake among the different dose AESG-administered groups relative to the Model group. Nevertheless, with the prolongation of AESG administration, the body weight of mice in all AESG dose groups was significantly lower than that in the Model group. This demonstrates that AESG exerts an inhibitory effect on HFD-induced body weight gain in mice. The results of fasting blood glucose (Figure 3C) showed that long-term HFD feeding elevated the fasting blood glucose level in the Model group. After AESG intervention, the fasting blood glucose level tended to decrease in all AESG-treated groups, with the AESG-M demonstrating the most pronounced efficacy.

HFD can induce fat accumulation in the body, affecting multiple organs. For instance, the liver, as a crucial organ for lipid metabolism, is responsible for the synthesis, decomposition, and transportation of lipids [20,25]. Adipose tissue, on the other hand, serves as the primary site for fat storage and also functions as a secretory organ that produces and releases various adipokines [26,27]. After HFD feeding, mice develop metabolic syndromes similar to those in humans, including obesity, insulin resistance, and fatty liver disease. For such metabolic disorders, the organs and adipose tissues of experimental animals are commonly used to evaluate the effects of HFD. These tissues can directly reflect in vivo metabolic changes and endocrine function, such as hepatic steatosis, adipocyte hypertrophy and hyperplasia, and inflammatory responses, making them essential indicators for assessing the efficacy of drug interventions.

Organ weight and organ coefficient are simple yet effective crucial indicators for evaluating the physical status of mice. By measuring the ratio of the weight of a specific organ to the body weight of mice, they can reflect the developmental status of the organ and the overall health condition of the organism [28], the study on the liver, spleen, and kidneys of mice revealed that compared with the Control group, the weights of the liver, spleen, and kidneys in the Model group were significantly increased (Figure 3D–F). This increase may be attributed to the following factors: high-fat diet induced hepatic lipid accumulation and inflammatory edema, compensatory hyperplasia of the spleen caused by metabolic inflammation, and lipotoxicity and compensatory hypertrophy in the kidney, which together led to the elevation of organ wet weights. In contrast to the Model group, after administration of AESG and Ato, the organ weights showed a decreasing trend, among which the liver weight was significantly reduced. Further research on the organ coefficients of each group indicated that the liver coefficient in the Model group was significantly higher than that in the Control group, with a statistically significant difference. Compared with the Model group, the liver coefficients in all AESG dose groups and the Ato group showed a decreasing trend after intervention.

Adipose tissue plays a crucial role in metabolic pathological states induced by a HFD, and changes in adipose tissue directly reflect the metabolic status of the organism [29,30]. Further studies were conducted on epididymal WAT and scapular BAT of mice. Compared with the Control group, the WAT weight and WAT index in the Model group were extremely significantly increased (### *p* < 0.001). In contrast to the Model group, the WAT weight and WAT index in all drug-administered groups showed a decreasing trend. After intervention with medium and high doses of AESG, the WAT weight was significantly reduced (** *p* < 0.01), and the difference in WAT index was statistically significant (Figure 3G). Compared with the Control group, the BAT weight and BAT index in the Model group were relatively increased. This may be an attempt by the organism to counteract the effects of partial fat accumulation by enhancing the activity of BAT. When compared with the Model group, the BAT index in all drug-administered groups increased to varying degrees, especially in the low-dose AESG group where the BAT was significantly increased (* *p* < 0.05) (Figure 3H). These results suggest that AESG may be involved in the metabolic transformation of adipose tissue and enhance the activity and energy metabolism of BAT.

### 3.3. Effect of AESG Intake on Tissue Steatosis

**Figure 4:**
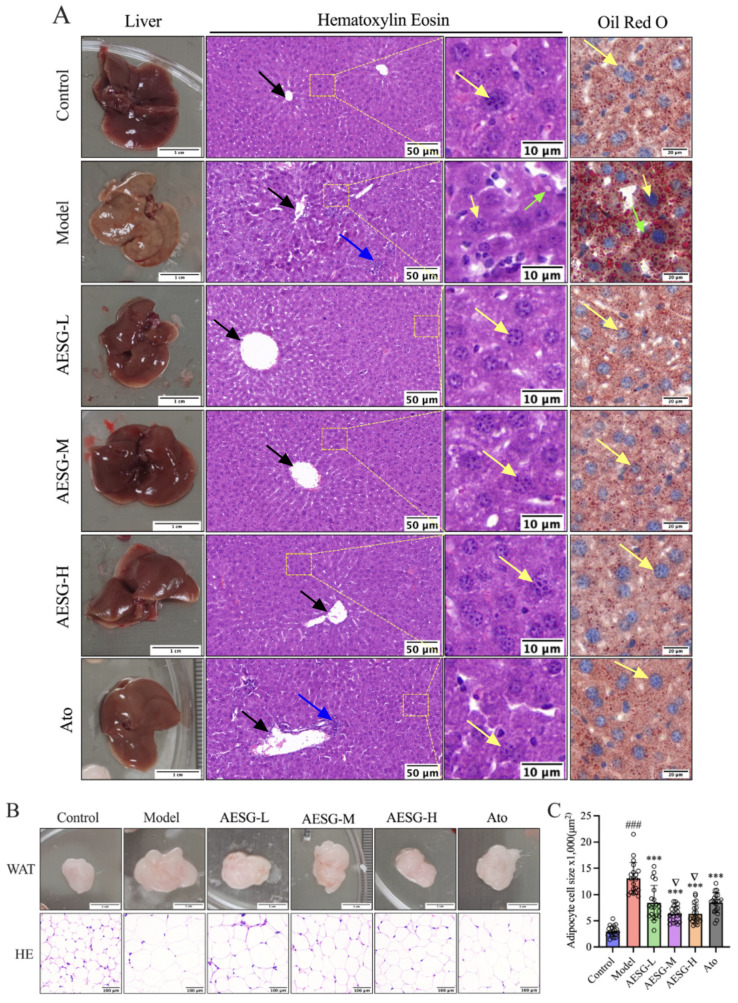
Effects of AESG intervention on histopathology of mice. (**A**) Gross morphology of liver tissue, and HE and ORO staining of liver tissue. Yellow arrows indicate the nuclei of hepatocytes; green arrows indicate lipid droplets; black arrows indicate central veins; blue arrows indicate focal infiltration of inflammatory cells. (**B**) Gross morphology and HE staining results of WAT. (**C**) AESG treatment reduces the size of white adipocytes in HFD fed mice, each circle represents one individual data point. Compared with the Control group, ### *p* < 0.001; Compared with the Model group, *** *p* < 0.001; Compared with the Ato group, ^∇^*p* < 0.05, with *n* = 20.

Long-term HFD feeding exerted a significant impact on the gross morphological features of mouse livers (Figure 4A). The livers in the Control group exhibited a ruddy color, firm texture, and clear, sharp edges, which were attributed to normal hepatocyte structure, minimal lipid accumulation, and normal blood perfusion under physiological conditions. In contrast, the livers in the Model group showed increased volume, tense capsule, pale creamy color, greasy texture, mottled parenchymal markings, and rounded edges. These changes were caused by excessive abnormal lipid accumulation in hepatocytes induced by long-term HFD feeding; lipid droplets filled the hepatocytes, causing the liver tissue to lose its normal ruddy color and present a pale, greasy pathological appearance. In the Ato-administered group, the degree of liver swelling was alleviated, but the overall color remained relatively pale. After intervention with low, medium, and high doses of AESG, the liver color gradually became ruddy, the texture recovered elasticity, and the degree of liver swelling was improved to varying extents.

HE staining is a commonly used method for observing morphological changes in the pathological structure of liver tissue, which is applied to investigate the causes, occurrence, and progression of lesions [31]. As observed from the HE staining results of liver tissue (Figure 4A), the hepatocytes in the Control group were radially arranged around the central vein, with abundant cytoplasm and large, round nuclei. After 8 weeks of HFD induction, bridging necrosis of hepatocytes between the central veins and portal areas was observed in the liver tissue of the Model group. The hepatic cords were irregular, and round vacuoles of varying sizes (identified as lipid droplets, LDs) were present in the cytoplasm, accompanied by inflammatory cell infiltration. In the Ato-treated group, LDs accumulation was reduced, but inflammatory cell infiltration still persisted. Following intervention with low, medium, and high doses of AESG, the HFD-induced liver pathological changes were alleviated to varying degrees. The hepatic cell cords became distinct, the cytoplasm was plump, and no obvious inflammatory cell infiltration was detected.

During the preparation of HE-stained sections, organic solvents such as xylene and ethanol are required for processing. Lipids in tissues are highly soluble in organic reagents and thus easily eluted. Therefore, it is difficult to distinguish intracellular lipids using HE staining alone, necessitating the use of specific methods for identifying intracellular fats. ORO is a classic dye for labeling lipids in liver tissue [32,33]. Accordingly, ORO staining was performed on the liver tissues of mice in each group. Compared with the Control group, a large number of red LDs of varying sizes were deposited in the hepatocytes of the Model group, almost filling the entire cytoplasm. After intervention with AESG and Ato, the accumulation of LDs was significantly reduced (Figure 4A). These results indicate that AESG intervention can alleviate hepatic inflammatory cell infiltration induced by long-term HFD and reduce the accumulation of LDs in hepatocytes, thereby exerting the hepatoprotective and hepatic lipid-lowering effects of the AESG.

WAT primarily functions to store TG. The main structure of WAT cells consists of a large single LD that displaces the nucleus to the cell periphery (referred to as a peripheral nucleus), with a thin layer of cytoplasm surrounding the LD. Due to the massive storage of TG, WAT exhibits an overall white appearance. As observed from the gross morphology and HE staining results of WAT (Figure 4B), the WAT in the Control group was relatively small in overall size, with adipocytes that were small and contained LDs accounting for a low proportion of the cell volume. After HFD feeding, the adipocytes in the Model group showed characteristic increases in both cell volume and LD proportion. Following administration of AESG and Ato, a significant reduction in adipocyte volume was observed in all drug-treated groups. To further quantify these changes, ImageJ software(version 2.1.0/1.53c) was used for quantitative analysis. Twenty measurement points were selected from WAT sections of each group to determine the adipocyte area. Compared with the Control group, the adipocyte area in the Model group was extremely significantly increased (### *p* < 0.001, Figure 4C). In contrast to the Model group, the adipocyte area in the WAT of all drug-administered groups was extremely significantly reduced (*** *p* < 0.001). Additionally, compared with the Ato group, the adipocyte area in the WAT of the medium and high-dose AESG groups was significantly decreased (^∇^*p* < 0.05). These results are consistent with the previously reported WAT index data of each group, indicating that AESG can improve the phenomena of significantly increased adipocyte size and LD proportion in the WAT of mice induced by long-term HFD feeding.

### 3.4. Effect of AESG Consumption on Plasma and Hepatic Lipid Levels in Mice

**Figure 5:**
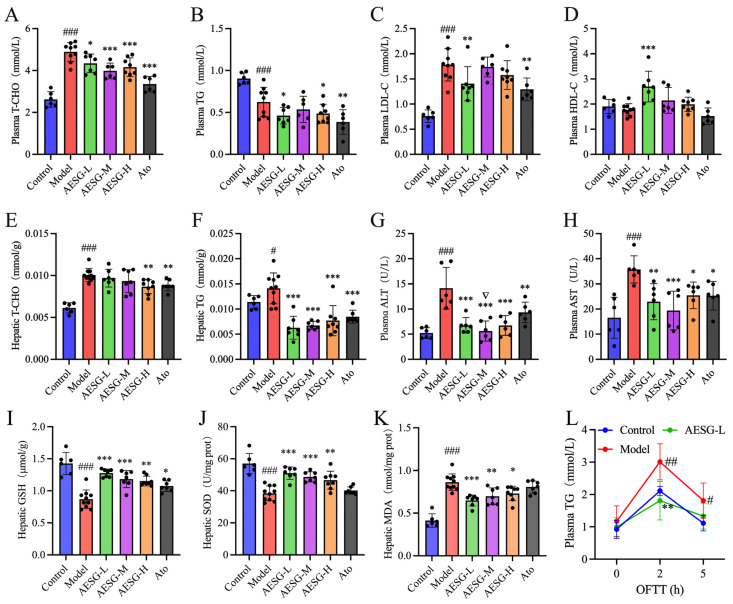
Effects of AESG intervention on biochemical indicators and oral fat tolerance in HFD-Fed Mice. (**A**) Plasma T-CHO level. (**B**) Plasma TG level. (**C**) Plasma LDL-C level. (**D**) Plasma HDL-C level. (**E**) Hepatic T-CHO content. (**F**) Hepatic TG content. (**G**) Plasma ALT level. (**H**) Plasma AST level. (**I**) Hepatic GSH content. (**J**) Hepatic SOD content. (**K**) Hepatic MDA content. (**L**) The result of oral fat tolerance test. Compared with the Control group, # *p* < 0.05, ## *p* < 0.01, ### *p* < 0.001; compared with the Model group, * *p* < 0.05, ** *p* < 0.01, *** *p* < 0.001; compared with the Ato group, ^∇^*p* < 0.05.

Compared with the Control group, the plasma levels of T-CHO and LDL-C in the Model group were extremely significantly increased (### *p* < 0.001). In contrast to the Model group, the plasma T-CHO levels were significantly reduced in the medium and high-dose AESG groups (*** *p* < 0.001), and the plasma LDL-C content was significantly decreased in the AESG-L group (** *p* < 0.01). After administration of AESG, the plasma TG levels were lower than those in the Model group, while the HDL-C levels were higher. Particularly, the plasma HDL-C level in the AESG-L group was extremely significantly higher than that in the Model group (*** *p* < 0.001). These results indicate that AESG can significantly improve dyslipidemia induced by HFD (Figure 5A–D).

The contents of T-CHO and TG in the liver tissue of the Model group were significantly higher than those in the Control group (Figure 5E,F). This indicates that long-term HFD not only leads to elevated blood glucose and dyslipidemia but also induces hepatic lipid accumulation. Compared with the Model group, both Ato and AESG-H significantly reduced the hepatic T-CHO content (** *p* < 0.01). The hepatic TG levels were extremely significantly decreased in the Ato group and all AESG groups (*** *p* < 0.001). Notably, the hepatic TG content was 0.0141 mmol/g in the model group, which was markedly decreased to 0.0063 mmol/g in the low-dose AESG group, with a reduction rate of 55.00%. Additionally, the hepatic lipid-lowering effect of AESG showed a tendency to be superior to that of Ato.

### 3.5. Effect of AESG Consumption on Hepatic Enzyme in HFD-Fed Mice

Liver function impairment induced by long-term HFD is a common pathological state in mice with MAFLD. ALT and AST are two commonly used biomarkers for evaluating liver function, and elevated levels of these enzymes typically indicate hepatocellular injury [34,35]. Compared with the Control group, the plasma levels of ALT and AST in the Model group were extremely significantly increased (### *p* < 0.001, Figure 5G,H). This indicates that long-term HFD feeding induced hepatic steatosis and impaired liver function in mice. In contrast to the Model group, the plasma contents of ALT and AST were significantly reduced after intervention with AESG and Ato. Notably, the medium-dose AESG group exhibited the most significant reduction (*** *p* < 0.001), which was significantly superior to that of the Ato group (^∇^*p* < 0.05). These findings suggest that the AESG not only reduces hepatic lipid accumulation but also exerts a hepatoprotective effect.

### 3.6. Effect of AESG Intervention on Antioxidant Capacity in HFD-Fed Mice

In the pathological state of MAFLD, oxidative stress serves as a core pathological mechanism. Hepatic GSH, SOD and MDA can reflect the systemic reserve of reducing substances, antioxidant capacity, and lipid peroxidation level, respectively. Compared with the Control group, the GSH level and SOD activity in the liver tissue of the Model group were significantly decreased, while the MDA level was significantly increased, indicating aggravated oxidative stress and reduced antioxidant capacity in the local liver tissue. Compared with the Model group, intervention with low-, medium-, and high-dose AESG increased GSH level and SOD activity and decreased MDA level to varying degrees, among which the low-dose effect was better. This suggests that AESG may improve MAFLD by enhancing antioxidant capacity (Figure 5I–K). Based on previous reports, mogrosides derived from *Siraitia grosvenorii* possess antioxidant activity [36]; in addition to mogrosides, AESG also contains polyphenolic components such as curcumin, which have been shown to exert antioxidant activity [37]. Therefore, these components are likely to act synergistically to mediate the antioxidant activity of AESG.

### 3.7. Effect of AESG Consumption on Lipid Absorption in HFD-Fed Mice

Oral fat tolerance test (OFTT) can be used to evaluate the absorption, transport, and clearance of exogenous fat in vivo. In this study, high-fat diet successfully induced metabolic dysfunction in mice, as evidenced by significantly higher serum TG peak in the model group than in the control group, suggesting impaired lipid clearance and exacerbated systemic lipid metabolism disorder in model mice. After AESG intervention, the TG peak was significantly reduced, indicating that AESG can improve fat tolerance and promote lipid clearance (Figure 5L).

### 3.8. AESG Reshaped the Gut Microbiota Profiles in HFD-Fed Mice

According to the dynamic monitoring results of blood lipids during animal experiments and the effect of AESG on hepatic TG levels in model mice, the low-dose AESG group showed the most pronounced lipid-lowering trend. Based on this, six biological replicates of cecal content samples were randomly selected from each of the control, model, and AESG-L groups for 16S rRNA gene sequencing, aiming to systematically evaluate the regulatory effect of AESG on the gut microbiota of MAFLD mice.

**Figure 6:**
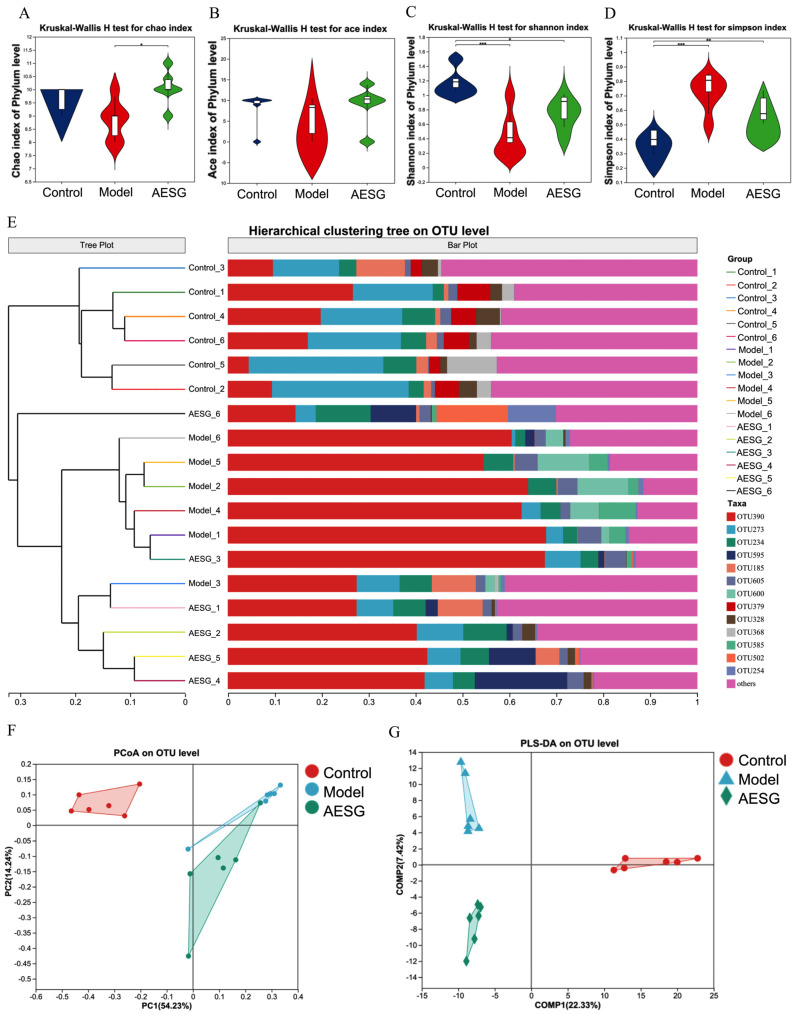
Improvement effect of AESG on gut microbiota diversity in MAFLD mice. (**A**–**D**) represent the alpha diversity of intestinal microbiota, including the Chao, Ace, Shannon, and Simpson indices, respectively. * *p* < 0.05, ** *p* < 0.01, *** *p* < 0.001. (**E**) Hierarchical clustering analysis of intestinal microbiota beta diversity at the OTU level. (**F**) PCoA of intestinal microbiota beta diversity. (**G**) PLS-DA of intestinal microbiota beta diversity. In (**F**,**G**), dots of different colors or shapes represent sample groups under different environments or conditions, with X-axis and Y-axis scales as relative distances having no actual physical meaning.

To evaluate the overall richness and diversity of the gut microbiota, we calculated and compared the alpha diversity indices among the Control, Model, and AESG groups. These indices included the Chao1 index and ace index, which reflect species richness (Figure 6A,B), as well as the Shannon index and Simpson index, which characterize both species evenness and richness (Figure 6C,D). Compared with the Control group, the Chao1 index and ace index were decreased in the Model group, while the Shannon index was extremely significantly reduced (*** *p* < 0.001) and the Simpson index was extremely significantly increased (*** *p* < 0.001). These results indicate that a HFD not only reduced the number of microbial species but also altered the evenness of the community structure, leading to increased community dominance and decreased diversity. However, after AESG intervention, compared with the Model group, the Chao1, ace, and Shannon indices were all elevated, while the Simpson index was decreased. This demonstrates that AESG intervention can effectively restore the HFD-induced reductions in gut microbiota richness and diversity. To assess the effects of HFD and AESG intervention on the overall community structure of the gut microbiota, Beta diversity analysis was performed. Consistent results from hierarchical clustering analysis, principal coordinate analysis (PCoA), and partial least squares discriminant analysis (PLS-DA) showed significant separation in the gut microbiota structures among the three groups (Figure 6E–G). Furthermore, AESG intervention could redirect the disrupted microbiota structure toward that of the Control group. These three complementary analyses collectively reveal that HFD induces profound disruption of the overall gut microbiota structure, and AESG administration can effectively modulate this disrupted state.

**Figure 7:**
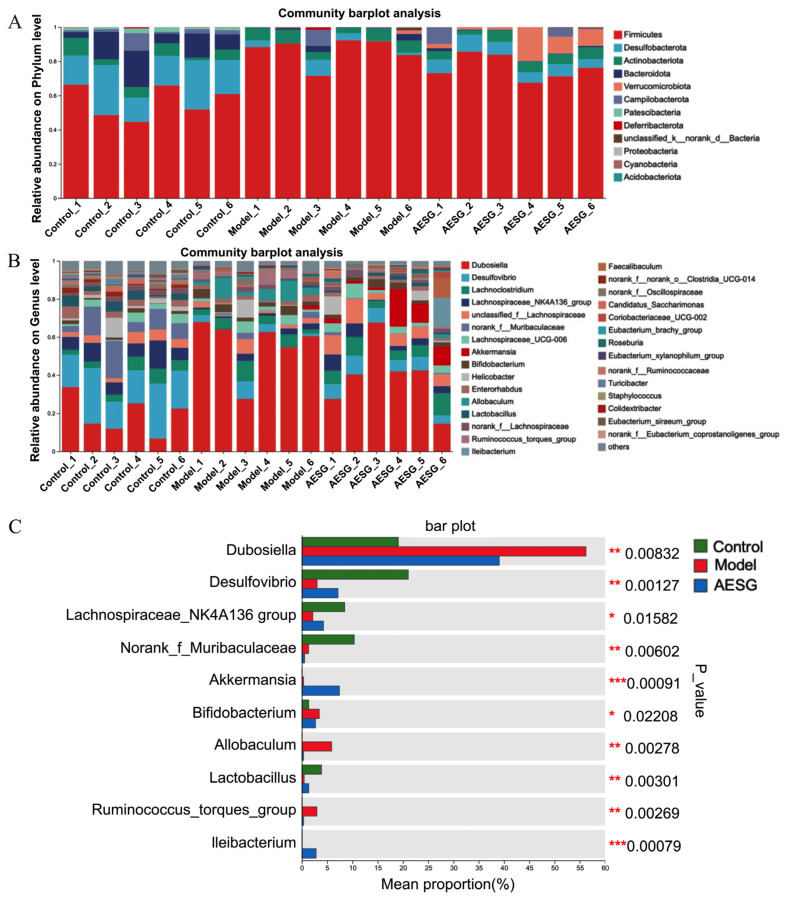
Microbial community composition profiling and genus-level multi-group comparison. (**A**,**B**) show the relative abundance of community composition at the phylum and genus levels, respectively. (**C**) Multi-group difference test at the genus level. Multi-group comparisons: * *p* < 0.05, ** *p* < 0.01, *** *p* < 0.001.

Through the analysis of gut microbiota community structure, we found that a HFD significantly altered the composition of the mouse gut microbiota at both the phylum and genus levels (Figure 7A,B), while AESG intervention effectively reversed most of the adverse changes induced by HFD. At the phylum level, the gut microbiota in all groups was predominantly dominated by Firmicutes. Compared with the Control group, the Model group exhibited an increased relative abundance of Firmicutes and a decreased relative abundance of Bacteroidetes, which represents the most classic microbiota signature in obesity and related metabolic diseases [38,39,40]. After AESG intervention, the relative abundance of Firmicutes showed a decreasing trend, while the relative abundance of Verrucomicrobiota—a phylum that enhances intestinal barrier function—was increased. Verrucomicrobiota can degrade intestinal mucus, promote mucus layer renewal, and improve metabolism [41,42]. To more accurately identify the bacterial taxa with altered abundances, we further analyzed the microbiota composition at the genus level and performed a differential abundance analysis across multiple groups (Figure 7C). HFD led to the depletion of various beneficial bacteria, such as *Lactobacillus* and the *Lachnospiraceae* NK4A136 group [43]. Notably, unlike typical reports, the abundance of *Dubosiella* was increased in the HFD-induced Model group [44]. We hypothesize that this may be a compensatory response to specific components of the HFD; however, this single microbial change was insufficient to offset the overall metabolic disorders caused by HFD. AESG intervention specifically increased the abundance of *Akkermansia*, a bacterium widely recognized as a guardian of intestinal barrier function and systemic metabolic health [45]. The elevated abundance of *Akkermansia* provides strong evidence for the superior efficacy of AESG in shaping a healthy gut microbiota.

**Figure 8:**
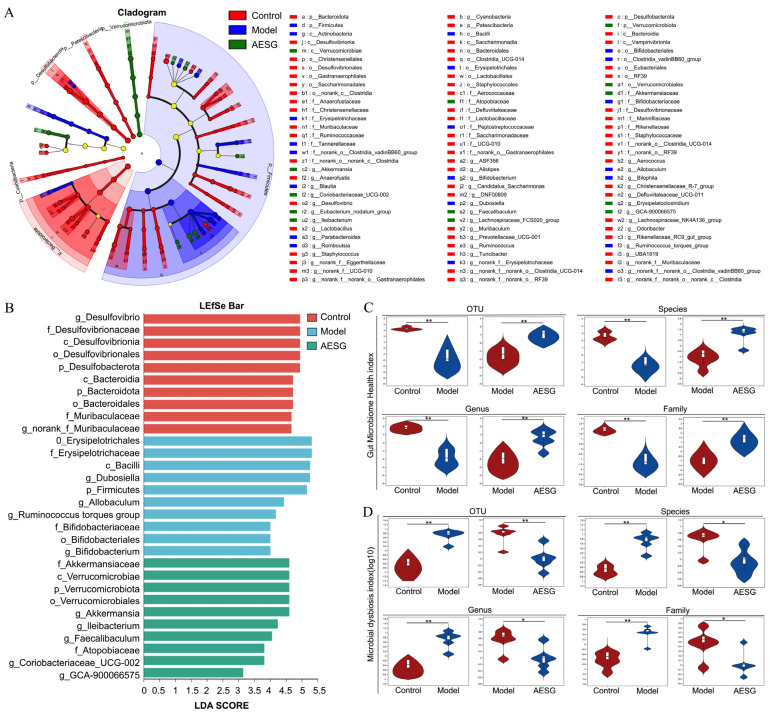
LEfSe analysis outcomes and gut microbiota health and dysbiosis indexes across OTU, Species, Genus and Family levels. (**A**) Species cladogram of LEfSe analysis. (**B**) LDA result table of LEfSe analysis. (**C**) Gut microbiome health index at the OTU, Species, Genus, and Family levels. (**D**) Intestinal microbial dysbiosis index at the OTU, Species, Genus, and Family levels. Pairwise Comparisons: * *p* < 0.05, ** *p* < 0.01.

To identify the key biomarkers that can best distinguish the microbial community structures among the three groups, we performed Linear Discriminant Analysis Effect Size (LEfSe) analysis. The species cladogram (Figure 8A) clearly illustrates the taxa with significant inter-group differences at all taxonomic levels ranging from phylum to genus. By integrating the Linear Discriminant Analysis (LDA) discriminant result graph (Figure 8B), which lists the top 10 biomarkers along with their LDA scores and corresponding groups, we were able to quantify the discriminative power of these biomarkers. The blank group exhibited a healthy microbial community structure. Bacteria such as those belonging to the Muribaculaceae family and the Bacteroidota phylum are efficient specialists in dietary fiber degradation. They can ferment complex carbohydrates to produce short-chain fatty acids, which supply energy to the host and maintain the acidic environment of the intestinal tract, thereby forming a stable and metabolically active foundational microecosystem [46,47,48]. The microbial characteristics of the model group revealed complex dysbiosis induced by a HFD Among these characteristics, the significant proliferation of the Ruminococcus_torques_group is of greater pathological significance. The overgrowth of this bacterial group directly impairs the integrity of the intestinal mucosal barrier, increases intestinal permeability, and serves as a key factor driving systemic low-grade inflammation and metabolic disorders [49]. The microbial biomarkers in the AESG-administered group indicated a distinct therapeutic ecological structure. The most prominent feature was the specific enrichment of *Akkermansia*, accompanied by an increase in *Faecalibaculum*, a bacterium that produces short-chain fatty acids. This finding demonstrates that AESG intervention can synergize with various beneficial bacteria to jointly establish a healthy ecological environment centered on barrier protection and metabolic regulation, which confirms the therapeutic potential of AESG from a microbiological perspective.

To evaluate the overall health status of the microbial community at the functional level, we calculated the gut microbiome health index and microbial dysbiosis index across multiple taxonomic levels (OTU, Species, Genus, Family) (Figure 8C,D). Compared with the Control group, the Model group showed a significant reduction in the health index and a marked elevation in the dysbiosis index at all four taxonomic levels. This quantitatively confirms the severe deterioration of the microbial ecosystem induced by HFD. After AESG intervention, the health index of the administration group was significantly increased at all levels, while the dysbiosis index was notably decreased, with values restored to a level close to that of the Control group. This result strongly demonstrates that AESG can not only alter the abundance of specific microbial taxa but also fundamentally restore the overall health and stability of the intestinal microecosystem.

### 3.9. Effects of AESG on FFA-Induced HepG2 Cells

**Figure 9:**
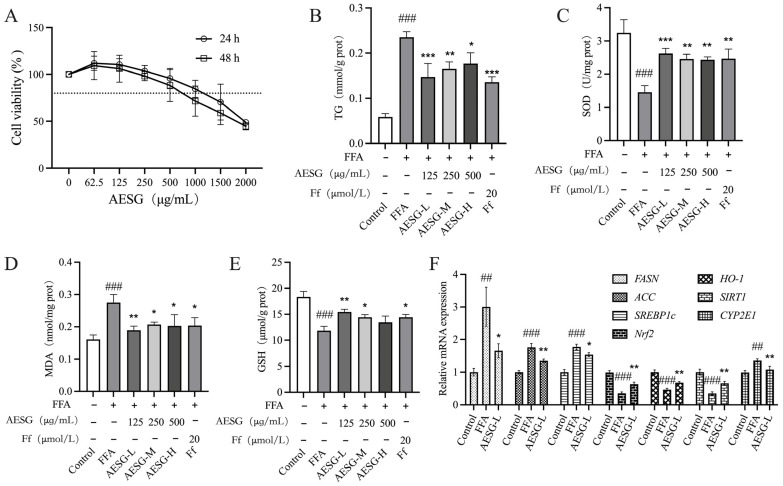
Effects of AESG on HepG2 cells. (**A**) Effects of different concentrations of AESG on cell viability, the dotted line represents the 80% cell viability line. (**B**) Intracellular TG content. (**C**) Intracellular SOD activity. (**D**) Intracellular MDA levels. (**E**) Intracellular GSH levels. (**F**) Effects of AESG on the mRNA expression of MAFLD-related genes in FFA-induced HepG2 cells. Compared with the Control, ## *p* < 0.01, ### *p* < 0.001. Compared with the FFA, * *p* < 0.05, ** *p* < 0.01, *** *p* < 0.001.

The Cell Counting Kit-8 (CCK-8) assay was employed to evaluate the effect of AESG on the viability of HepG2 cells. Concentrations that retained more than 80% of cell viability were selected as the optimal administration concentrations; ultimately, 125, 250, and 500 μg/mL were designated as the low-, medium-, and high-dose AESG groups, respectively (Figure 9A). Compared with the Control group, intracellular TG content was significantly increased in the FFA group. In contrast, intervention with either AESG or Ff remarkably reduced intracellular TG accumulation to varying extents when compared with the FFA group (Figure 9B), indicating a potential lipid-lowering effect of AESG.

To comprehensively assess the oxidative stress status and disease progression of MAFLD, the levels of SOD, MDA, and GSH were detected in combination. Relative to the Control group, the FFA group exhibited a significant decrease in SOD activity, a notable increase in MDA levels, and a marked reduction in GSH content. These results demonstrated that FFA exposure could induce impairment of the antioxidant defense system in HepG2 cells. Notably, AESG intervention significantly reversed these abnormal changes: SOD activity was dramatically increased, MDA levels were substantially decreased, and GSH content was effectively restored, suggesting that AESG exerts potent antioxidant activities in HepG2 cells (Figure 9C–E). Furthermore, this study explored the regulatory effect of AESG-L on the mRNA expression levels of genes associated with FFA-induced MAFLD in HepG2 cells (Figure 9F). Compared with the FFA group, AESG-L treatment significantly downregulated the mRNA expression of fatty acid synthesis-related genes (*FASN*, *ACC*, and *SREBP1c*) and the oxidative stress-related gene *CYP2E1*, while concurrently upregulating the expression of antioxidant-related genes (*Nrf2*, *HO-1*, and *SIRT1*). Collectively, these findings imply that AESG may alleviate the pathological progression of MAFLD by suppressing de novo lipid synthesis and enhancing cellular antioxidant capacity in FFA-induced HepG2 cells.

## 4. Discussion

MAFLD is a systemic metabolic disease closely associated with obesity, insulin resistance and dyslipidemia, and its global prevalence has brought severe challenges to public health. At present, lifestyle intervention is the first-line treatment for MAFLD, but its poor long-term compliance limits clinical application, and there is an urgent need to explore safe and effective natural intervention strategies. *Siraitia grosvenorii* is a typical edible and medicinal plant in China, and its water extract is the most consistent with traditional folk usage. This study systematically confirmed the therapeutic effect of AESG on MAFLD through in vivo and in vitro experiments, and revealed its multi-target regulatory mechanism, which fills the gap in the research on the holistic effect of AESG on MAFLD.

The anti-obesity and metabolic regulatory effects of AESG are the core basis of its MAFLD improvement. In this study, AESG significantly inhibited HFD-induced body weight gain in mice without affecting food intake, which indicated that the weight loss effect of AESG was not caused by appetite suppression but by regulating energy metabolism and lipid deposition—a finding superior to the positive drug atorvastatin, which reduced body weight by decreasing food intake. Traditionally, adipose tissue was regarded as a passive lipid storage depot. However, a growing body of research has revealed that adipose tissue is an active metabolic organ [50], playing a crucial role in regulating the body’s energy homeostasis [51]. Further analysis of adipose tissue found that AESG reduced the weight and volume of WAT and increased the index of BAT. BAT is a key thermogenic tissue that can consume lipids to produce heat, and its activation is closely related to the improvement of insulin resistance and hepatic steatosis. The regulation of AESG on adipose tissue suggests that it may promote the browning of WAT or the activation of BAT, thereby increasing energy expenditure and reducing systemic lipid accumulation [52], which is an important mechanism for its amelioration of MAFLD. In addition, AESG effectively reduced fasting blood glucose and improved plasma lipid disorders in MAFLD mice [53,54], which further confirmed its regulatory effect on glucose and lipid metabolism, and laid a foundation for improving the core metabolic abnormalities of MAFLD.

The hepatoprotective effect of AESG is reflected in the reduction in hepatic lipid deposition and the improvement of liver function and pathological damage. Hepatic steatosis is the initial pathological feature of MAFLD, and the accumulation of triglycerides in hepatocytes is the core manifestation. This study found that AESG significantly reduced the content of T-CHO and TG in the liver of MAFLD mice, and Oil Red O staining further confirmed the reduction in intracellular lipid droplets. Meanwhile, AESG significantly lowered the plasma levels of ALT and AST—the classic biomarkers of hepatocellular injury, indicating its good hepatoprotective activity. Histopathological results showed that AESG alleviated HFD-induced hepatic cord disorder, hepatocyte necrosis and inflammatory cell infiltration, which was consistent with the improvement of liver function indices. It is worth noting that the plasma TG level of C57BL/6J mice in the model group showed a decreasing trend, the result was consistent with the report by Pan et al. [55], which is related to the genetic characteristics of this strain with strong lipid metabolism adaptability [56], while the significant increase in hepatic TG indicated that hepatic lipid deposition is an earlier pathological change than blood lipid abnormality in HFD-induced MAFLD [57]; AESG can effectively reverse this early damage, which is of great significance for the early intervention of MAFLD.

Oxidative stress is a key link in the progression of MAFLD from simple steatosis to steatohepatitis; the imbalance of the antioxidant system in the liver is an important pathological basis. This study found that HFD significantly reduced the levels of GSH and SOD in the liver of mice and increased the content of MDA—the product of lipid peroxidation—while AESG intervention reversed these abnormal changes, especially the low-dose AESG showed a better antioxidant effect. Given the diverse chemical composition of AESG, which contains polyphenolic components such as curcumin in addition to mogrosides, it is plausible that these compounds collectively contribute to the observed antioxidant effects through synergistic interactions. Consistent with previous reports, polyphenolic bioactive compounds are known to effectively reduce MDA levels and enhance the activities of antioxidant enzymes such as SOD, thereby alleviating oxidative stress [37]. In vitro experiments further confirmed that AESG increased the activity of SOD and the content of GSH in FFA-induced HepG2 cells, reduced the level of MDA, and regulated the expression of oxidative stress-related genes: down-regulating *CYP2E1* (a key enzyme promoting reactive oxygen species production) and up-regulating the *Nrf2*/*HO-1*/*SIRT1* signaling pathway (the core antioxidant defense system of cells). These results indicate that AESG can enhance the antioxidant capacity of the liver and cells, reduce oxidative stress damage, and thus inhibit the pathological progression of MAFLD. In addition, the oral fat tolerance test showed that AESG reduced the plasm TG peak of MAFLD mice, indicating that it can improve the absorption and clearance capacity of exogenous fat, reduce the lipid load of the liver, and further alleviate hepatic lipid deposition.

The detection results of gut microbiota provide key clues for understanding the mechanism by which AESG improves MAFLD. A high-fat diet induces a complex and imbalanced microbiota structure: although it unexpectedly enriches *Dubosiella*, it leads to the loss of core functional bacteria such as Lactobacillus and the *Lachnospiraceae* NK4A136 group, ultimately resulting in metabolic disorders. In contrast, AESG intervention exhibits a superior regulatory capacity that transcends the impact of diet. It not only synergistically increases the abundance of multiple beneficial bacteria (e.g., *Dubosiella* and *Akkermansia*) but also fully restores the *Lachnospiraceae* NK4A136 group—a key functional bacterium lost due to the high-fat diet—while maintaining the inhibition of harmful bacteria. This multi-targeted, synergistic microbiota remodeling effect is the core mechanism underlying AESG’s therapeutic efficacy. As a plant extract, the saponin components of AESG are not easily absorbed by the upper gastrointestinal tract and can reach the intestine directly to act as prebiotics, selectively promoting the growth of beneficial bacteria. A healthy gut microbiota can produce beneficial metabolites such as short-chain fatty acids, thereby alleviating hepatic steatosis through the “gut-liver axis”—a critical pathway [58].

The material basis of the pharmacological effects of AESG is its diverse chemical components, and this study tentatively identified 38 components including tetracyclic triterpenoid saponins, flavonoids and phenylpropanoids, with tetracyclic triterpenoid saponins (mogrosides) as the main constituent. The multi-component characteristics of AESG enable it to exert a synergistic effect through multiple pathways, which is the advantage of natural plant extracts over single chemical drugs.

This study still has some shortcomings and limitations. First, although low-, medium-, and high-dose AESG groups were set up, the results of multiple tests did not show a dose-dependent effect. This may be attributed to the relatively small number of mice in each group or significant individual differences among the mice. Second, the effect of AESG on microbial metabolites such as short-chain fatty acids was not detected, and the specific signal transmission pathway of the gut-liver axis needs to be further clarified. Third, the in vitro experiment only used the HepG2 cell line, and the verification of primary hepatocytes or organoid models is needed to improve the reliability of the results. Future research can carry out fecal microbiota transplantation experiments to directly verify the role of gut microbiota in the therapeutic effect of AESG; use transcriptomics, proteomics and metabolomics to explore the key molecular targets and signaling pathways of AESG; conduct pathway inhibition or gene silencing experiments to further clarify the molecular mechanism; and separate and purify the active components of AESG to clarify their individual and synergistic effects, so as to provide a more detailed scientific basis for the development of *Siraitia grosvenorii* as a MAFLD intervention product.

## 5. Conclusions

In summary, this study confirmed that AESG has a significant therapeutic effect on HFD-induced MAFLD in mice, and that its mechanism is multi-faceted and multi-targeted. First, it regulates glucose and lipid metabolism, inhibits body weight gain, reduces fasting blood glucose, and improves plasma dyslipidemia. Second, it alleviates hepatic lipid deposition, reduces hepatocellular injury, and improves liver function and pathological damage of liver and adipose tissues. Third, it enhances the antioxidant capacity of the liver and cells, reducing oxidative stress and lipid peroxidation damage by regulating the *Nrf2*/*HO-1*/*SIRT1* signaling pathway. Fourth, it remodels the balance of gut microbiota, restoring the richness and diversity of intestinal flora, enriching beneficial bacteria such as *Akkermansia*, inhibiting the overgrowth of harmful bacteria, and maintaining gut homeostasis to play a role through the gut-liver axis.

This study systematically revealed the therapeutic effect and mechanism of AESG on MAFLD from in vivo to in vitro, from organism to cellular and molecular levels; it also clarified the material basis of its efficacy with tetracyclic triterpenoid saponins as the main constituent. The research results confirm the potential of *Siraitia grosvenorii* as a natural, edible, and medicinal resource for MAFLD intervention, breaking through the limitation of previous studies focusing on a single component and highlighting the advantages of the holistic effect of AESG consistent with traditional usage. As a safe and non-toxic natural extract, AESG provides a promising dietary intervention strategy for the prevention and auxiliary treatment of MAFLD, and lays a solid scientific foundation for the development and utilization of *Siraitia grosvenorii* as a functional food or natural medicine for metabolic disease intervention. In the future, in-depth research on the optimal dose, active components and specific molecular targets of AESG will further promote its clinical transformation and application in the field of MAFLD prevention and treatment.

## Data Availability

The original contributions presented in this study are included in the article/Supplementary Materials. Further inquiries can be directed to the corresponding authors.

## Supplementary Materials

The following supporting information can be downloaded at: https://www.mdpi.com/article/10.3390/foods15071241/s1, Supplementary methods; Table S1: Gradient Elution Conditions; Table S2: cDNA Synthesis System 1; Table S3: cDNA Synthesis System 2; Table S4: RT-qPCR primer sequences; Table S5: RT-qPCR reaction system; Table S6: RT-qPCR reaction procedure; Table S7: Identification of Chemical Constituents in AESG.

## Abbreviations

The following abbreviations are used in this manuscript:

AESG	Aqueous Extract of *Siraitia grosvenorii*
MAFLD	Metabolic dysfunction-associated fatty liver disease
LHG	Luo Han Guo
HFD	high-fat diet
T-CHO	Total cholesterol
TG	Triglyceride
LDL-C	Low-density lipoprotein cholesterol
HDL-C	High-density lipoprotein cholesterol
ALT	Alanine aminotransferase
AST	Aspartate aminotransferase
Ato	Atorvastatin
WAT	white adipose tissue
BAT	brown adipose tissue
GSH	Glutathione
SOD	Superoxide dismutase
MDA	Malondialdehyde
*ACC*	Acetyl-CoA Carboxylase
*FASN*	Fatty Acid Synthase
*HO-1*	Heme Oxygenase 1
*GAPDH*	Glyceraldehyde-3-Phosphate Dehydrogenase
*Nrf2*	Nuclear Factor Erythroid 2-Related Factor 2
*SREBP1c*	Sterol Regulatory Element-Binding Protein 1c
*CYP2E1*	Cytochrome P450 Family 2 Subfamily E Member 1
*SIRT1*	Sirtuin 1
