# Supplementary material for: Aqueous Extract of Siraitia grosvenorii Alleviates MAFLD by Modulating Metabolism and Maintaining Gut Homeostasis in High-Fat Diet Fed Mice
Source: Foods. 2026 Apr 5;15(7):1241. doi: 10.3390/foods15071241 (PMC13072755; doi:10.3390/foods15071241)
Supplement: Supplementary file 1 [file foods-15-01241-s001.zip › foods-4192762-supplementary.pdf]

Aqueous extract of *Siraitia grosvenorii* alleviates MAFLD by modulating metabolism and maintaining gut homeostasis in high-fat diet fed mice

Supplementary Methods

1. Gut Microbiota Analysis

DNA Extraction: Microbial genomic DNA was extracted from samples of different groups using the FastPure Stool DNA Isolation Kit (MJYH, Shanghai, China) according to the manufacturer's instructions. The integrity of the extracted genomic DNA was verified by 1% agarose gel electrophoresis, and the DNA concentration and purity were determined using a NanoDrop 2000 spectrophotometer (Thermo Scientific, USA).

PCR Amplification and Sequencing Library Construction: The extracted DNA was used as the template for PCR amplification of the V3–V4 hypervariable region of the 16S rRNA gene. The upstream primer 338F (5′-ACTCCTACGGGAGGCAGCAG-3′) and downstream primer 806R (5′-GGACTACHVGGGTWTCTAAT-3′) with barcode sequences were used. The PCR mixture (20 μL) contained: 5 × TransStart FastPfu buffer (4 μL), 2.5 mM dNTPs (2 μL), 5 μM forward primer (0.8 μL), 5 μM reverse primer (0.8 μL), TransStart FastPfu DNA polymerase (0.4 μL), and template DNA (10 ng). PCR amplification was performed as follows: initial denaturation at 95 °C for 3 min; 27 cycles of denaturation at 95 °C for 30 s, annealing at 55 °C for 30 s, and extension at 72 °C for 30 s; a final extension at 72 °C for 10 min; and holding at 4 °C (ABI GeneAmp® 9700 thermal cycler). PCR products were recovered using 2% agarose gel electrophoresis and purified with a PCR Clean-Up Kit (Yuhua, China). Purified amplicons were quantified using a Qubit 4.0 fluorometer (Thermo Fisher Scientific, USA). Sequencing libraries were generated using the NEXTFLEX Rapid DNA-Seq Kit: (1) adapter ligation; (2) removal of adapter self-ligated fragments by magnetic bead purification; (3) library enrichment by PCR amplification; (4) final library recovery using magnetic beads. Sequencing was performed on the Illumina Nextseq 2000 platform (Shanghai Majorbio Bio-pharm Technology Co., Ltd., China).

High-Throughput Sequencing Data Analysis: Raw paired-end reads were quality-controlled using fastp (version 0.19.6, https://github.com/OpenGene/fastp) and merged using FLASH (version 1.2.11, http://www.cbcb.umd.edu/software/flash). The optimized sequences were denoised using the DADA2 plugin in the QIIME2 pipeline with default parameters to generate amplicon sequence variants (ASVs). To minimize the influence of sequencing depth on downstream alpha and beta diversity analyses, all samples were rarefied to 20,000 sequences. After rarefaction, the average Good's coverage of all samples remained at 99.09%. Taxonomic annotation of ASVs was performed using the Naive Bayes classifier in QIIME2 against the SILVA 16S rRNA database (v138).

Statistical Analysis: All statistical analyses were performed on the Majorbio Cloud Platform (https://cloud.majorbio.com). Alpha diversity indices (Chao 1, Shannon, etc.) were calculated using mothur (version 1.30.2, http://www.mothur.org/wiki/Calculators). Between-group differences in diversity were analyzed using the Wilcoxon rank-sum test. Principal coordinate analysis (PCoA) based on bray–curtis distance was performed to evaluate the similarity of microbial community structures among samples. LEfSe (Linear discriminant analysis Effect Size, http://huttenhower.sph.harvard.edu/LEfSe) was used to identify bacterial

taxa with significantly different abundances from phylum to genus level among groups (LDA score > 2, $p < 0.05$).

2. HepG2 Cell experiments

2.1. Solution Preparation

Preparation of complete DMED medium: The basal DMEM medium, fetal bovine serum (FBS), and double-antibody solution were mixed uniformly at a volume ratio of 89:10:1.

Preparation of 20% BSA (bovine serum albumin): 6 g of fatty acid-free BSA was accurately weighed and transferred into a 50 mL centrifuge tube. Phosphate buffer solution (PBS) 30 mL was slowly added, followed by centrifugation at 8000 rpm for 20 min. The resulting clear solution was obtained as the final product.

Preparation of sodium oleate stock solution: 0.1218 g of sodium oleate was accurately weighed into a 50 mL centrifuge tube. 10 mL of ultrapure water was slowly added, mixed, and dissolved in a water bath at 70 °C until completely dissolved, yielding a 40 mM sodium oleate stock solution. Then, 10 mL of the prepared 20% BSA solution was added to the dissolved sodium oleate stock solution, mixed well, and filtered through a 0.22 μm sterile filter to obtain a 20 mM sodium oleate stock solution. The solution was aliquoted and stored at 4 °C for further use.

Preparation of sodium palmitate stock solution: 0.05568 g of sodium palmitate was accurately weighed and placed in a 50 mL centrifuge tube. 10 mL of ultrapure water was slowly added, mixed thoroughly, and dissolved in a water bath at 70 °C until completely dissolved, yielding a 20 mM sodium palmitate stock solution. Then, 10 mL of the prepared 20% BSA solution was added to the dissolved sodium palmitate stock solution, mixed well, and filtered through a 0.22 μm sterile filter to obtain a 10 mM sodium palmitate stock solution. The solution was aliquoted and stored at 4 °C for further use.

2.2. Cell Culture (using T25 flasks as an example)

Cell Resuscitation：The cryopreservation tube containing cells was removed from liquid nitrogen (caution: wear an explosion-proof tube mask) and immediately thawed in a 37 °C water bath to ensure complete melting within 1 min with no visible crystals. The outer surface of the cryotube was wiped with 75% ethanol before being transferred into a biological safety cabinet. The cell suspension was transferred to a 15 mL centrifuge tube containing 5 mL of complete DMEM medium, followed by centrifugation at 1000 rpm for 5 min. The supernatant was discarded, and the cell pellet was resuspended in 5 mL of complete medium, then inoculated into a T25 culture flask. The cells were cultured in a cell incubator at 37 °C with 5% CO_2 and 95% air. The culture medium was replaced with fresh complete medium the next day for continued culture.

Cell Passaging：When cells reached more than 80% confluence, the culture medium in the T25 flask was discarded, and cells were washed twice with PBS, followed by removal of PBS. 1 mL of 0.25% trypsin solution pre-warmed in a 37 °C water bath was added. Cells were observed under an inverted microscope until they retracted and rounded up. Then, complete medium with a volume three times that of the trypsin was added to terminate digestion. Cells were gently detached from the bottom of the flask by pipetting, and the cell suspension was transferred to a 15 mL centrifuge tube, followed by centrifugation at 1000 rpm for 5 min. The supernatant was discarded, and the cell pellet was resuspended in 1–2 mL of fresh complete

medium. Cells were subcultured at a ratio of 1:2, gently mixed, and incubated in the cell incubator for further culture.

2.3. Determination of Alterations in Gene Expression Levels via RT-qPCR

RNA Extraction from Cells: The cell culture medium was removed, and cells were washed twice with sterile PBS. Trizol lysis reagent was added directly to the culture plate at a ratio of 1 mL per 10 cm^2, and cells were lysed completely by repeated pipetting. The lysate was transferred to a 1.5 mL RNase-free centrifuge tube, stored in a low-temperature freezer for 5 min, mixed thoroughly by vortexing, and this step was repeated 1–3 times. The lysate was then kept at room temperature for 5–10 min to allow complete separation of nucleoproteins and nucleic acids. Chloroform was added at a volume of 1/5 of the Trizol volume, and the tube was shaken vigorously by hand for about 15 s until the solution turned milky white. After standing at room temperature for 3 min, the mixture was centrifuged at 12000 rpm for 15 min at 4 °C in a pre-cooled centrifuge. After centrifugation, the mixture separated into three layers: the upper aqueous phase containing RNA, and the middle and lower organic phases. The upper aqueous phase was carefully collected (without aspirating the middle or lower organic phases to avoid genomic DNA contamination) and transferred to a new RNase-free centrifuge tube. An equal volume of isopropanol was added, mixed gently by inversion, and kept at −20 °C for 10 min, followed by standing at room temperature for 15 min. The sample was centrifuged at 12000 rpm for 10 min at 4 °C. After centrifugation, a gelatinous RNA precipitate was visible on the tube wall or bottom. The supernatant was discarded, and residual liquid at the tube mouth was blotted with filter paper. An appropriate volume of 75% ethanol prepared with DEPC-treated water was added, and the precipitate was washed gently by inversion. After standing at room temperature for 5 min, the sample was centrifuged at 7500 rpm for 5 min at 4 °C. The supernatant was discarded, and the RNA precipitate was dried at room temperature for 5–10 min. Excessive drying should be avoided, as it will make RNA difficult to dissolve. The RNA was dissolved thoroughly in 30 μL of RNase-free ddH_2O. The RNA concentration was determined using a NanoDrop 2000 Ultra-Micro Spectrophotometer (Thermo Fisher Scientific). RNA purity was assessed according to the absorbance ratio at 260 nm and 280 nm; a ratio of OD_{260}/OD_{280} between 1.8 and 2.0 was regarded as acceptable purity. RNA samples were stored at −80 °C or used directly for subsequent experiments.

mRNA Reverse Transcription：Total RNA (1 μg) from each sample was used as the template for reverse transcription. According to the measured RNA concentration, the volume of each reagent required for a 10 μL reaction system was calculated for each sample. All components of the reverse transcription kit were thawed on ice and briefly centrifuged. Reagents for cDNA Synthesis System 1 were sequentially added into sterile, nuclease-free PCR tubes according to Table S2. The mixture was gently mixed by pipetting, briefly centrifuged, incubated at 37 °C for 2 min, and then incubated at 65 °C for 2 min. The reaction mixture was placed on ice and briefly centrifuged. Subsequently, the required reagents were sequentially added according to Table S3. The mixture was gently mixed by pipetting, briefly centrifuged, and incubated at 50 °C for 15 min. After the reaction, it was incubated at 85 °C for 5 min to terminate the reaction. The resulting cDNA solution was placed on ice and used directly for subsequent experiments or stored at −20 °C for further use.

RT-qPCR Analysis: The sequences of human-specific primers are shown in Table S4. Primers were diluted to a working concentration of 10 μM with DEPC-treated water. Reaction reagents were sequentially added according to the qPCR reaction system shown in Table S5. Each group included 3 biological replicates, and each sample was run in 3 technical replicates. The mixture was gently mixed by pipetting. After sample loading, the plate was sealed, briefly

centrifuged, and placed in a quantitative real-time PCR instrument. Amplification was performed using the reaction program set in Table S6, and the Ct values were finally obtained.

Table S1. Gradient Elution Conditions

Time（min）	Flow Rate（mL/min）	A（%）	B（%）
0	0.4	80	20
5	0.4	78	22
12	0.4	73	27
17	0.4	65	35
20	0.4	50	50
27	0.4	80	20
32	0.4	80	20

Table S2. cDNA Synthesis System 1

Reagent	Usage amount
Template RNA	1 μg（Convert to volume according to the concentration）
10×DNase buffer	1 μL
dsDNase	1 μL
Nuclease-Free Water	To 10 μL

Table S3. cDNA Synthesis System 2

Reagent	Usage amount
Product of cDNASynthesisSystem1	10 μL
All-in-One First-Strand Synthesis Master Mix	4 μL
Nuclease-Free Water	To 20 μL

Table S4. RT-qPCR primer sequences

Gene name	F（5'-3'）	R（5'-3'）
GAPDH	AGAAGGCTGGGGCTCATTTG	AGGGGCCATCCACAGTCTTC
FASN	GGATCACAGGGACAACCTGG	GGGAGATGAGGGGAGTTCCT
ACC	AATAGCGTCTCTAACTTCCTTCAC	CCGTCACTCAGCCGATGTA
SREBP1c	TCTGGGTTTTGTGTCTTCAGC	GGAGGTGAGAAGGGACAACTG
SIRT1	GGTCCCCAATCACCTCATCTG	TGCACAGGGATGTTCCAGTTC
NRF2	GGCATCACCAGAACACTCAG	TGACCAGGACTTACAGGCAAT
HO-1	TCTTGGCTGGCTTCCTTACC	GGATGTGCTTTTCGTTGGGG
CYP2E1	CGCTGCTGGACTACAAGGA	GGAAGAGGATGTCGGCTATGA

Table S5. RT-qPCR reaction system

Reagent	Volume	Final concentration
Taq SYBR Green qPCR Premix	10 μL	1×
Forward primer（10 μM）	0.4 μL	0.2 μM
Reverse primer（10 μM）	0.4 μL	0.2 μM
DNA template	X μL	10～200 ng/20 μL
Nuclease-Free Water	To 20 μL	-

Table S6. RT-qPCR reaction procedure

Reaction process	Reaction temperature（°C）	Reaction time	Cycle number
Pre-denaturation	95	30 s	1
Denaturation	95	10 s	40
Annealing	60	10 s	40
Extension	72	30 s	40
Termination reaction	4	-	1

Table S7. Identification of Chemical Constituents in AESG

No.	Compound Name	CAS	Retention Time(min)	Ion Type	Measured *m/z*	Theoretical*m/z*	Absolute Error	Molecular Formula	MS²	Compound Class
1	Coumarin	91-64-5	4.06	$[M+H]^+$	147.0441	147.0446	-0.0005	$C_9H_6O_2$	147, 146, 128, 110, 81, 70	Phenylpropanoid
2	Vanillin	121-33-5	5.31	$[M-H]^-$	151.0392	151.0395	-0.00033	$C_8H_8O_3$	151, 136, 107, 71	Aromatic Aldehyde
3	n-Nonanoic acid	112-05-0	15.54	$[M-H]^-$	157.1227	157.1229	-0.00022	$C_9H_{18}O_2$	158, 130, 116, 89, 59	Fatty Acid
4	9-Decenoic acid	14436-32-9	25.05	$[M+H]^+$	171.1376	171.1385	-0.00091	$C_{10}H_{18}O_2$		Fatty Acid
5	Myristaldehyde	104-67-6	3.05	$[M+H]^+$	181.4977	181.0501	0.4476	$C_9H_8O_4$	180, 152, 134, 127, 108, 85, 69	Lactone
6	Heptyl isobutyrate	2349-13-5	3.18	$[M-H]^-$	185.1652	185.1542	0.01104	$C_{11}H_{22}O_2$		Lactone
7	2,4-Diacetoxypentane	7371-86-0	2.05	$[M+H]^+$	189.1234	189.1127	0.01074	$C_9H_{16}O_4$	189, 143, 132, 86	Lactone
8	Damascone	23726-91-2	12.28	$[M+H]^+$	191.1435	191.1436	-0.00012	$C_{13}H_{20}O$		Monoterpene
9	2-Tridecanone	593-08-8	2.45	$[M+H]^+$	199.1807	199.2062	-0.02552	$C_{13}H_{26}O$		Aliphatic Ketone
10	Lauric acid	143-07-7	11.34	$[M+H]^+$	201.1641	201.1855	-0.02139	$C_{12}H_{24}O_2$		Fatty Acid
11	1-Acetyl-β-carboline	50892-83-6	14.29	$[M+H]^+$	211.0869	211.0871	-0.00020	$C_{13}H_{10}N_2O$	211, 193, 169	Alkaloid
12	Ar-turmerone	532-65-0	1.27	$[M-H]^-$	215.1399	215.1436	-0.00371	$C_{15}H_{20}O$	215, 171, 116, 69	Sesquiterpene
13	N-Phenyl-2-naphthylamine	135-88-6	1.828	$[M+H]^+$	220.1181	220.1226	-0.00447	$C_{16}H_{13}N$	220, 173, 132, 90, 86, 60	Aromatic Amine
14	Bis-(5-formylfurfuryl)	7389-38-0	7.76	$[M+H]^+$	235.0605	235.0606	-0.00013	$C_{12}H_{10}O_5$		Ether

	ether									
15	Magnolol	528-43-8	2.21	$[M+H]^+$	267.1341	267.1385	-0.00441	$C_{18}H_{18}O_2$	267, 249, 189, 121, 108, 96, 81	Phenylpropanoid
16	Methyl palmitate	112-39-0	25.91	$[M+H]^+$	271.2636	271.2637	-0.00012	$C_{17}H_{34}O_2$		Lactone
17	1-Butyl 2-isobutyl phthalate	17851-53-5	22.58	$[M+H]^+$	279.1592	279.1596	-0.00040	$C_{16}H_{22}O_4$	279， 205, 149, 57	Lactone
18	Diisobutyl phthalate	84-69-5	19.38	$[M+H]^+$	279.1592	279.1596	-0.00040	$C_{16}H_{22}O_4$		Lactone
19	Terephthalic acid	100-21-0	6.56	$[M+H]^+$	279.1595	279.1596	-0.00012	$C_{16}H_{22}O_4$		Aromatic Hydrocarbon
20	9-Octadecenoic acid (Z)-, sulfurized	68412-07-7	23.974	$[M-H]^-$	281.2489	281.2481	0.00080	$C_{18}H_{34}O_2$		Fatty Acid
21	Hexadecyl acetate	629-70-9	23.32	$[M-H]^-$	283.2647	283.2637	0.00098	$C_{18}H_{36}O_2$	283, 214, 70	Lactone
22	Kaempferol	520-18-3	9.36	$[M+H]^+$	287.0553	287.0556	-0.00033	$C_{15}H_{10}O_6$		Flavonoid
23	Corydaline	100041-05-2	10.73	$[M+H]^+$	309.0871	309.0875	-0.00043	$C_{17}H_{12}N_2O_4$	309, 281, 263, 206, 207, 71	Alkaloid
24	Curcumin	458-37-7	8.13	$[M+H]^+$	369.1313	369.1338	-0.00254	$C_{21}H_{20}O_6$		Aromatic Phenol
25	Dioctyl phthalate	117-84-0	20.44	$[M+H]^+$	391.2845	391.2848	-0.00028	$C_{24}H_{38}O_4$		Lactone
26	β-Sitosterol	83-46-5	26.11	$[M+H]^+$	415.3892	415.394	-0.00484	$C_{29}H_{50}O$	415, 338, 172, 119, 69	Steroid
27	Kaempferol-7-O-rhamnoside	20196-89-8	9.22	$[M+H]^+$	433.1136	433.1135	0.00009	$C_{21}H_{20}O_{10}$	433, 287, 86	Flavonol Glycoside
28	Mogrol	88930-15-8	16.46	$[M+H]^+$	475.3788	475.3787	0.00005	$C_{30}H_{52}O_4$		Tetracyclic Triterpene
29	Kaempferitrin	482-38-2	7.47	$[M+H]^+$	579.1714	579.1714	-0.00001	$C_{27}H_{30}O_{14}$	433, 287, 85, 71	Flavonoid
30	Grosvenorine	156980-60-8	6.98	$[M+H]^+$	741.2243	741.2242	0.0001	$C_{33}H_{40}O_{19}$	596, 433, 287, 129,	Flavonoid

									85	
31	Mogroside II-A1	88901-44-4	13.71	$[M+H]^+$	801.4959	801.5	-0.00409	$C_{42}H_{72}O_{14}$	603, 459, 441, 423, 173, 119, 95, 85	Tetracyclic Triterpene
32	Mogroside IIE	88901-38-6	13.25	$[M+H]^+$	801.4988	801.5	-0.00122	$C_{42}H_{72}O_{14}$	603, 441, 423, 121, 95, 85	Tetracyclic Triterpene
33	Mogroside III	130567-83-8	11.87	$[M+H]^+$	963.5525	963.5529	-0.00041	$C_{48}H_{82}O_{19}$	603, 441, 423, 121, 95, 85	Tetracyclic Triterpene
34	Mogroside IV	89590-95-4	10.404	$[M+H]^+$	1125.606	1125.606	0.00001	$C_{54}H_{92}O_{24}$	603, 459, 441, 423, 145, 127, 85	Tetracyclic Triterpene
35	Siamenoside I	126105-12-2	11.34	$[M+H]^+$	1125.606	1125.606	0.00001	$C_{54}H_{92}O_{24}$	603, 459, 441, 423, 325, 187, 145, 127, 85	Tetracyclic Triterpene
36	11-O-Mogroside V	126105-11-1	9.99	$[M-H]^-$	1283.629	1283.627	0.00183	$C_{60}H_{100}O_{29}$	1283, 1121, 797, 101, 113	Tetracyclic Triterpene
37	Mogroside V	88901-36-4	11.08	$[M-H]^-$	1285.644	1285.643	0.00114	$C_{60}H_{102}O_{29}$	1285, 1123, 800, 221, 113, 101	Tetracyclic Triterpene
38	Mogroside VI	89590-98-7	9.48	$[M+H]^+$	1449.713	1449.711	0.00171	$C_{66}H_{112}O_{34}$	603, 585, 441, 423, 325, 145, 127	Tetracyclic Triterpene
